# Digital Interventions for Improving Body Dissatisfaction in Children and Emerging Adults: Systematic Review and Meta-Analysis

**DOI:** 10.2196/72231

**Published:** 2025-08-13

**Authors:** Li Liu, Jianning Yang, Fengmei Tan, Xia Yang, Huan Luo, Yanhua Chen, Xiaolei Zhao

**Affiliations:** 1 School of Nursing, Southwest Medical University, 1 Xianglin Road, Longmatan District, Luzhou, Sichuan, 646000, China, 86 13980250224; 2 Department of Hematopathology, The Affiliated Hospital, Southwest Medical University, Luzhou, Sichuan, China; 3 Department of Nephrology, The Affiliated Hospital, Southwest Medical University, Luzhou, Sichuan, China; 4 Department of Nursing, The Affiliated Hospital, Southwest Medical University, Luzhou, Sichuan, China

**Keywords:** body dissatisfaction, digital intervention, children, emerging adults, systematic review, meta-analysis, PRISMA

## Abstract

**Background:**

Body dissatisfaction is a condition where individuals are dissatisfied with their physical appearance. It has become a global issue, especially among children and emerging adults. A growing number of digital interventions have been developed to address body dissatisfaction in children and emerging adults; however, controversies remain regarding their efficacy, underscoring the need for a comprehensive synthesis of current evidence.

**Objective:**

This systematic review aimed to explore the effectiveness of digital interventions in improving body image–related outcomes among children and emerging adults.

**Methods:**

From inception to April 24, 2024, a literature search was performed across 7 databases—PubMed, Web of Science, MEDLINE, EBSCO (Elton B Stephens Company), Cochrane Library, CNKI (China National Knowledge Infrastructure), and WANFANG—to identify randomized controlled trials (RCTs) with a predefined set of inclusion criteria. This systematic review was reported in line with PRISMA (Preferred Reporting Items for Systematic Reviews and Meta-analyses) guidelines. Study selection, data extraction, and risk of bias assessment using the Cochrane Risk-of-Bias Tool 2.0 were conducted independently by 2 researchers. Standardized mean differences (SMDs) and 95% CIs from the included RCTs were calculated for the meta-analysis. Heterogeneity was assessed with *I*² values. A fixed-effects model was used when *I*²≤50%, and a random-effects model was selected when *I*²>50%.

**Results:**

Twenty RCTs with 5251 participants (2610 in intervention groups and 2641 in control groups) met the inclusion criteria. Digital interventions included web pages, mobile apps, computer-based videos, computer-based sessions, internet-based sessions, internet games, chatbots, podcasts, and social media. Our results indicate that digital interventions could significantly improve body dissatisfaction (SMD=0.38, 95% CI −0.63 to −0.13; *I*^2^=55%; *P*=.003), physical appearance comparison (SMD=−0.24, 95% CI −0.45 to −0.03; *I*^2^=0%; *P*=.003), thin-ideal internalization (SMD=−0.28, 95% CI −0.36 to −0.2; *I*^2^=41%; *P*<.001), self-esteem (SMD=0.14, 95% CI 0.07-0.21; *I*^2^=21%; *P*<.001), self-compassion (SMD=0.55, 95% CI 0.33-0.78; *I^2^*=35%; *P*<.001), and depression (SMD=−0.59, 95% CI −0.97 to −0.21; *I*^2^=0%; *P*=.002), with small to medium effect sizes.

**Conclusions:**

While digital interventions improved body dissatisfaction among children and emerging adults, additional well-designed, rigorous, and large-scale RCTs are needed to decisively provide estimates of the effectiveness of digital interventions on body dissatisfaction.

## Introduction

### Prevalence and Impacts of Body Dissatisfaction

Body image encompasses an individual’s cognition, emotional attitudes, and behavioral regulation regarding their body, and is a multidimensional concept [1]. Dissatisfaction with one’s body’s appearance, including its shape, weight, and other features, is known as body dissatisfaction [2]. Body dissatisfaction has a pervasive hold on children and emerging adults, and the phenomenon has been described as “normative discontent” [3]. Studies showed the prevalence of body dissatisfaction ranged from 42.2% to 80.9% among children and emerging adults across the world [4-7]. Females, those with higher BMI, and those from minority groups were reported to experience greater body dissatisfaction [8]. Body dissatisfaction can lead to a range of physiological issues, such as low self-esteem [9], anxiety [10], and depression [11], as well as behavioral burdens, such as eating disorders [12], extreme weight loss behaviors [13], alcohol, drug abuse [14], and excessive pursuit of cosmetic procedures [15]. The direct economic costs of body dissatisfaction were estimated to be US $84 billion in the United States [16].

### Existing Face-to-Face Interventions and Limitations

Existing face-to-face interventions, such as cognitive behavioral therapy (CBT) and behavior interventions and supports (BIS), demonstrated small to moderate efficacy in reducing body dissatisfaction. For instance, CBT helps reframe maladaptive thoughts about body image [17], while dissonance-based interventions like the EVERYbody Project reduced thin-ideal internalization and eating disorder symptoms in college students [18]. A systematic review revealed that behavior interventions and supports improved disordered eating, body dissatisfaction, and extreme weight control behaviors in girls, but not boys [19]. However, the face-to-face interventions are sometimes constrained by accessibility, the costly nature of the format, a global shortage of mental health professionals, or stigma [20].

### Efficacy of Digital Interventions

Digital technologies offered cost-effective alternatives in body dissatisfaction interventions [21]. Although studies identified that social media engagement was associated with higher body dissatisfaction and restricting food [22], digital interventions could offer especially critical support in adolescents [23]. A systematic review assessed the effectiveness of universal body image interventions delivered through a digital platform among young women. Most articles indicated that these interventions were effective in improving at least one body image outcome [24]. However, findings in other studies remained inconsistent. A study involving 127 girls aged 10-13 years, using videos developed by the Dove Self-Esteem Project, revealed that after intervention, there was no significant difference between the intervention group and the control group on body satisfaction [25]. This highlights the need for a comprehensive synthesis of current evidence.

### Gaps in Current Evidence

There was no systematic review to explore the effectiveness of digital interventions on body dissatisfaction in children and emerging adults through quantitative synthesis. In this regard, this study aimed to fill this identified gap in the literature through a meta-analysis and to synthesize the effectiveness of various digital interventions on children and emerging adults with body dissatisfaction. By providing meta-analyses of currently available randomized controlled trials in children and emerging adults, this paper aimed to consolidate evidence on the use of digital interventions to treat body dissatisfaction.

## Methods

### Search Strategy

This systematic review was reported in line with PRISMA (Preferred Reporting Items for Systematic Review and Meta-analysis) guidelines [26] (the PRISMA checklist is provided in Checklist 1). PubMed, Web of Science, MEDLINE, EBSCO (Elton B Stephens Company), Cochrane Library, the Chinese databases CNKI (China National Knowledge Infrastructure) and WANFANG were searched from inception to April 24, 2024. This process was carried out independently by 2 researchers (LL & JNY). The PICOS (population, intervention, comparison, outcome, and study design) formula was used:

- Population (#1): youth OR young* OR child* OR adolescent OR teen* OR juvenile OR junior OR girl OR boy OR adult or students;
- Intervention (#2): remote OR website OR digital OR online OR network OR phone OR internet OR eHealth OR mHealth OR app OR multimedia OR social media OR zoom OR Facebook OR Instagram OR telephone OR (virtual reality);
- Comparison (#3): “usual care”;
- Outcomes (#4): (body image) OR (body shape) OR (body dissatisfaction) OR (body weight) OR (physical appearance);
- Study design (#5): randomized controlled trials.

The final search strategy is #1 AND #2 AND #3, with #4 (Type of Study: randomized controlled trial) applied as a filter. Reference lists of publications were also searched for potentially relevant studies (see Multimedia Appendix 1).

### Inclusion and Exclusion Criteria

#### Design

Any randomized controlled trial (RCT) in English or Chinese that explored the effects of digital interventions on body dissatisfaction among children or emerging adults was included, including randomized waitlist-controlled trials and crossover RCTs.

#### Participants

Children (<18 years [27]) or emerging adults (18‐25 years [28]) with body dissatisfaction were included. Some special groups of children or emerging adults were excluded, including but not limited to those who were pregnant, postpartum women, new mothers, children, or emerging adults with amputation, patients with cancer, models, or cosmetic surgery sequelae. Only children’s data were extracted if parents and children had been involved in the RCTs as participants.

#### Intervention

Intervention methods were based on Internet or smartphone technologies, including apps, web pages, virtual reality, telemedicine, Zoom (Zoom Video Communications), Facebook (Meta Platforms), Instagram (Meta Platforms), and other social media.

#### Outcomes

Outcomes of interest include at least one of the following measures, primary outcomes: body image satisfaction or dissatisfaction, such as body dissatisfaction, body appreciation, shape and weight concerns, and physical appearance comparisons; secondary outcomes: internalized outcomes, such as self-esteem, self-compassion, thin-ideal internalization, and self-objectification; negative affect, such as depression and anxiety symptoms, and negative affect; and eating behaviors, such as eating disorder and eating restraint. These secondary outcomes were selected based on the Tripartite Influence Model [29], which presents that family, peers, and media influence an individual’s body dissatisfaction via appearance-related social comparisons and thin-ideal internalization. Thin-ideal internalization serves as a predictor of body dissatisfaction [30], while studies showed that body dissatisfaction acted as a risk factor for negative affect [31] and eating disorder pathology [32].

#### Study Selection

The reference manager EndNote 21 (Clarivate) was used to manage studies and remove duplicates. The remaining records were screened by 2 independent reviewers (LL and JNY) based on the titles and the abstracts. The full text of studies that either reviewer identified as potentially eligible continued to be read and screened by the 2 independent reviewers (LL and JNY) based on the inclusion and exclusion criteria. Any discrepancies were resolved with the third reviewer (XLZ) until consensus was reached.

#### Quality Assessment

The risk of bias in eligible RCTs was independently assessed by the 2 reviewers (LL and JNY) according to 7 domains of the Cochrane risk-of-bias tool for randomized trials [33]: (1) random sequence generation, (2) allocation concealment, (3) blinding of participants and personnel, (4) blinding of outcome assessment, (5) incomplete outcome data, (6) selective reporting, and (7) other potential sources of bias. For each domain, the risk of bias was classified as low, high, or unclear. Discrepancies were adjudicated by the third reviewer (XLZ) until a consensus was achieved. Studies were considered low risk of bias if all 7 domains were assessed as low risk, or only one domain was assessed as high risk or unclear. If 2 domains were assessed as high risk or unclear, the studies were determined to have some concerns. If more than 2 domains were assessed as high risk or unclear, the study was rated a high risk of bias [34].

#### Data Extraction

A standardized data extraction form was used to extract data from each study, which included the following details: (1) first author, the year of publication, and country; (2) inclusion and exclusion criteria; (3) sample size of the experimental and control groups; (4) intervention characteristics (contents of intervention and comparison, follow-up); and (5) outcomes and main results. Data were extracted by LL and JNY and verified by XLZ. Only the data in the first period were extracted in the randomized waitlist-controlled trials. Only the data in the digital intervention groups and the control groups were extracted when participants were divided into more than 2 groups.

#### Data Analysis

Review Manager v5.4.1 (Cochrane Collaboration) was used for data synthesis. The findings that could not be synthesized were narratively described based on the outcomes of interest. For the continuous variables, standardized mean difference (SMD) and 95% CIs were calculated through random or fixed-effects models when the studies assessed the same outcome. SMD values of 0.2‐0.5 represented a small effect size, 0.5‐0.8 was considered medium, and values greater than 0.8 were interpreted as large [35]. The effect size of individual studies was mainly combined using the random effects model because of the different scales involved in assessing the same outcomes. The *I^2^* statistic was used to assess the heterogeneity across studies. *I*^*2*^ values of 25%, 50%, and 75% were considered as low, moderate, and high heterogeneity [36]. When *I*²≤50%, a fixed-effects model was selected; otherwise, a random-effects model was used [37]. Strategies for addressing heterogeneity included choosing a random or fixed effect model, excluding studies, or conducting subgroup analysis. The test level was *α*=.05, and *P*<.05 was considered to indicate statistical significance.

### Ethical Considerations

This systematic review is based on the synthesis of previously published studies and does not involve the collection of primary data directly from human participants. As such, formal ethical approval, including an institutional review board approval number, informed consent, and compensation, was not applicable. However, we have adhered to the ethical principles of research, including the accurate and transparent reporting of study findings. We have also ensured that all included studies were conducted in accordance with relevant ethical guidelines and regulations.

## Results

### Search Results

**Figure 1.**
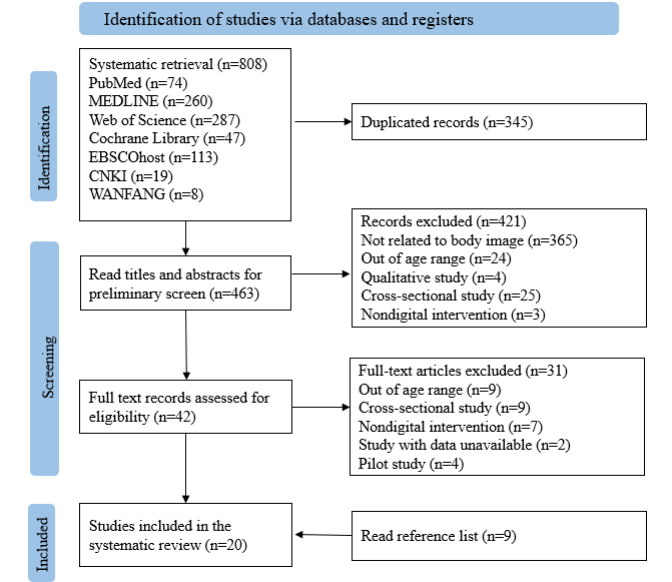
Preferred Reporting Items for Systematic Reviews and Meta-Analyses flow diagram.

A total of 808 records were identified from the initial literature search. After removing the duplicates, titles and abstracts were screened, and 42 studies were further reviewed in full text. Finally, 20 studies [25,38-56] met the inclusion criteria, including 9 [41-43,49,50,53-56] studies identified from reference lists. The study selection flow chart is shown in Figure 1.

### Study Characteristics

All the studies included were RCTs, including randomized waitlist-controlled trials (n=7). Only the data in the first phase were extracted in the randomized crossover and waitlist trials. Of the included studies, 20 RCTs with 5251 participants were included, including 2610 participants in the intervention groups and 2641 participants in the control groups. The studies were published between 2000 and 2023 and were conducted in the United States (n=10), Australia (n=3), the United Kingdom (n=3), China (n=1), Indonesia (n=1), Italy (n=1), and Brazil (n=1). Five studies [38-42] had mixed-gender samples, 15 [25,42-56] with only females. Sample sizes of the included studies ranged from 53 to 2000. All studies reported postintervention effects, whereas short-term and long-term follow-up data were reported in 11 [38,42,43,45,49,51-56] and 3 [39-41] studies, respectively. Participants and study characteristics are summarized in Table 1.

The digital interventions mainly included web page (n=6), computer-based sessions (n=1), internet-based sessions (n=2), computer-based videos (n=2), social media–based video (n=1), social media (n=3), such as Facebook and podcasts, mobile apps (n=2), internet games (n=1), animated films (n=1), and chatbot (n=1). Among the included studies, the interventions varied in length from 1 minute to 2 hours per session and in duration, ranging from a single day up to 8 weeks.

**Table 1.** Characteristics of the 20 included randomized controlled trials.

Author (year, country)	Participants	Sample size	Intervention	Comparison	Follow-up	Outcomes
[Bibr R38]Winzelberg et al,2000 (United States) [43][Bibr R38]	Female university students	T:^a^ n=31, C:^b^ n=29	Internet-based sessions	Waitlist control group	3 months	BSQ^c^, EDI-DT^d^, EDEQ^e^, WCSC^f^
[Bibr R39]Bruning et al, 2004 (United States) [44][Bibr R39]	14‐ to 16-year-old girls and their parents	T: n=102, C: n=51	Web page	Waitlist control group	6 months	WDEQ, WSCS^f^, EDI-DT
Low et al, 2006 (United States) [45][Bibr R40]	First- and second-year female undergraduates	T: n=14, C: n=14	Computer-Based sessions	No intervention	8 months	EDI^g^, SFRS^h^, EDI-DT, SATA^i^
[Bibr R41]Heinicke et al, 2007 (Australia) [46][Bibr R41]	12‐ to 18-year-old females	T: n=36, C: n=37	Internet-based sessions	Waitlist control group	2 months, 6 months	BSQ-SF^j^, BCS^k^, DEBQ^l^, SATA, BDI^m^
[Bibr R42]Cousineau et al, 2010 (United States) [38][Bibr R42]	6th-grade students	T: n=92, C: n=98	Web page	Science-based websites	3 months	BESAA^n^, SPPA^o^
Halliwell et al, 2011 (United Kingdom) [25]	10‐ to 13-year-old females	T: n=37, C: n=29	Computer-based videos	Newspaper or magazine	None	EDI-BD^p^, BISS^q^, BES^r^, DEBQ^l^, EDI-DT
Franko et al, 2012 (United States) [47][Bibr R43]	College women	T: n=32, C: n=32	Web page	Websites without information about eating	3 months	BSQ, SATAQ-3^s^
Stice et al, 2012 (United States) [48]	Female college students	T: n=19, C: n=20	Web page	Educational brochure group: brochure	None	BPS-SD^t^, IBSS^u^-R, DRES^v^, BDI, EDDI^w^
Serdar et al, 2014 (United States) [49]	18‐25 years old females	T: n=112, C: n=114	Web page	No intervention	8‐9 weeks	BES, EDDS^x^, IBSS-R^y^
Zhong et al, 2016 (China) [50]	Female college students	T: n=33, C: n=31	Web page	No intervention	None	BPS-SD, IBSS-R
Toole et al, 2016 (United States) [51]	18‐ to 21-year-old females	T: n=40, C: n=40	Social media	Waitlist control group	None	BSQ, SCS^z^, BAS^aa^, RSES^ab^, CSWS-AS^ac^
Slater et al,2017 (United Kingdom) [52]	8‐ to 9-year-old females	T: n=40, C: n=40	Internet games	Internet games without human figures	None	CFRS^ad^, MTST^ae^
Rodgers et al, 2018 (United States) [39]	14‐ to 18-year-old adolescents	T: n=129, C: n=123	Mobile app	No intervention	12 weeks	PNSC^af^, SCS^ag^, BESAA^ah^, PAC^ai^
[Bibr R50]Matheson et al, 2020 (United States) [40]	7‐ to 14-year-old children	T: n=442, C: n=446	Animated films	Animation without body image	None	VAS^aj^, CFRS
Seekis et al, 2020 (Australia) [53]	17‐ to 21-year-old females	T: n=42, C: n=34	Social media	Waitlist control group	1 month, 3 months	EDI-BD, EDI-DT, SAAS^ak^, UPACS^al^, BAS-2^am^, SCS-SF
Atkinson et al, 2021 (United Kingdom) [54]	Female undergraduates	T: n=67, C: n=65	computer-based videos	Documentary	1 week	VAS, BAS-2, BIAAQ^an^, SATAS^ao^, EDEQ, WSC^ap^
[Bibr R53]Cerea et al, 2021 (Italy) [41]	20‐ to 25-year-old females	T: n=25, C: n=25	Mobile app	Waitlist control group	16 days	QDC^aq^, DASS-21^ar^, EDI-3^as^
Garbett et al, 2023 (Indonesia) [55]	15‐ to 19-year-old females	T: n=924, C: n=923	Social media–based (Facebook and Instagram) videos	Waitlist control group	1 month	BESAA, SATAQ^at^, PNASC
[Bibr R55]Matheson et al, 2023 (Brazil) [42]	13‐ to 18-year-old Brazilian residents	T: n=355, C: n=443	Chatbot	Standard care	1 week, 1 month	BESAA, PNASC, BIS-ES^au^
Fardouly et al, 2023 (Australia) [56]	18‐ to 25-year-old females	T: n=38, C: n=47	Social media	Use Facebook as usual	4 weeks	EDI-BD, PNASC, BAS, PACS

a T: intervention group.

b C: control group.

c BSQ: Body Shape Questionnaire.

d EDI-DT: Eating Disorder Inventory-Drive for Thinness.

e EDEQ: Eating Disorder Examination Questionnaire.

f WCSC: Weight Concerns and Shape Concerns scale.

g EDI: Eating Disorders Inventory.

h SFRS: The Stunkard Figure Rating Scale.

i SATA: Sociocultural Attitudes Toward Appearance.

j BSQ-SF: Body Shape Questionnaire-Short form.

k BCS: Body Comparison Scale.

l DEBQ: Dutch Eating Behavior Questionnaire.

m BDI: Beck Depression Inventory.

n BESAA: Body Esteem Scale for Adolescents and Adults.

o SPPA: Self-Perception Profile for Adolescents

p EDI-BD: Body Dissatisfaction Subscale of the Eating Disorder Inventory.

q BISS: Body Image State Scale.

r BES: Body Esteem Scale.

s SATAQ-3: Sociocultural Attitudes Towards Appearance Questionnaire-3.

t BPS-SD: Satisfaction and Dissatisfaction with Body Parts Scale.

u IBSS: Ideal-Body Stereotype Scale-Revised.

v DRES: Dutch Restrained Eating Scale

w EDDI: Eating Disorder Diagnostic Interview.

x EDDS: Eating Disorder Diagnostic Scale.

y IBSS-R: Ideal-Body Stereotype Scale-Revised.

z SCS: Self-Compassion Scale.

aa BAS: Body Appreciation Scale.

ab RSES: Rosenberg Self-Esteem Scale.

ac CSWS-AS: Contingencies of Self-Worth Scale-Appearance Subscale.

ad CFRS: Child Figure Rating Scale.

ae MTST: Modified Twenty Statements Test.

af PNASC: Positive and Negative Affect Schedule for Children.

ag SCS-SF: Self-Compassion Scale-Short-Form.

ah BESAA: Body Esteem Scale for Adolescents and Adults.

ai PACS: Physical Appearance Comparison Scale.

aj VAS: visual analog scale.

ak SAAS: Social Appearance Anxiety Scale.

al UPACS: Upward Physical Appearance Comparison Scale.

am BAS-2: Body Appreciation Scale-2.

an BIAAQ: Body Image-Acceptance and Action Questionnaire.

ao SATAS: Sociocultural Attitudes Toward Appearance Scale.

ap WSC: Weight and Shape Concern.

aq QDC: Questionario sul Dismorfismo Corporeo.

ar DASS-21: Depression Anxiety Stress Scale-21.

as EDI-3: Eating Disorder Inventory-3.

at SATAQ: Sociocultural AttitudeTowardds Appearance Questionnaire.

au BIS-ES: Body Image Self-Efficacy Scale.

### Risk of Bias in Studies

Of the 20 included studies, only 4 (20%) were assessed as low risk of bias, while 13 (65%) demonstrated high risk of bias, and 3 (15%) were categorized as moderate risk of bias (see Multimedia Appendix 2).

#### Random Sequence Generation

This domain assesses whether the method used to generate the random sequence was adequate to prevent selection bias. Although all studies were reported to be RCTs, only 11 studies were assessed as having a low risk of bias in random sequence generation. Computer-generated random numbers were reported in 1 study [46], and a random number table was reported in 3 studies [25,39,42]. Cluster or block randomization was reported in 4 studies [38,41,53,55]. Minimization functions in Qualtrics were reported in 2 studies [40,56]. It was reported in 1 study [54] that whole timeslots were randomly allocated to a condition to avoid cross-contamination in an open computer laboratory, which was assessed as low risk of bias. However, 1 study [44] was assessed as having a high risk of bias in random sequence generation because students were assigned to a group based on class schedule rather than randomization. The remaining 8 studies [43,45,47-52], which did not mention a specific method used to generate the random sequence, were assessed as unclear bias.

#### Allocation Concealment

This domain evaluates whether the process of assigning participants to groups was concealed. Participants were reported to be randomly allocated to the intervention or control groups in all studies. Only 4 studies [39,41,49,53] reported the use of email to complete allocation concealment, which was assessed as a low risk of bias. In one study [55] reported participants and researchers were not concealed from the randomized arm, which was assessed as a high risk of bias. Due to a lack of further details about allocation concealment, 15 studies [25,38,40,42-48,50-52,54,56] were considered as an unclear risk of bias.

#### Blinding of Participants and Personnel

This domain assesses whether participants and researchers were blinded to group allocation. It was hard to achieve the blinding of participants or personnel because digital interventions were conducted in intervention groups and nondigital interventions were conducted in control groups in most studies involved. Internet-based or social media assessments and interventions were used in 6 studies [45,46,51,53-55], which may be interpreted as blinding of participants and personnel. Among the 6 studies, 1 study [45] reported that participants used a pseudonym. One study [54] reported that all assessments were self-reported anonymously via computer. One study [39] reported that assessments were housed on a survey software, and the procedure was identical for participants in both the intervention and control groups. Two studies [25,44] mentioned that participants were instructed not to discuss the study with other students to reduce potential cross-contamination, which might be seen as blinding participants and personnel. Four studies [40,42,47,49] were assessed as high risk of bias for their failure to blind participants. Seven studies [38,42,44,46,48,53,56] were assessed as unclear risk of bias due to a lack of further details being reported.

#### Blinding of Outcome Assessment

This domain evaluates whether the outcome assessors were blinded to the intervention group assignments. Twelve studies were assessed as having a low risk of bias in the blinding of outcome assessment. One [42] of these studies used dummy codes instead of participants’ names for outcome assessment. The outcome assessors in another 2 studies [48,55] were unaware of group allocation. Although assessors were not blinded, questionnaires were completed via website, social media, or application by participants in 6 studies [38,39,41,46,53,54]. Another 3 studies [47,49,56] were assessed as having a low risk of bias because questionnaires were sent by e-mail. Eight studies [25,38-40,46-48,50] were assessed as unclear due to the lack of clear information on whether the outcome assessors were blinded to the intervention group assignments. No study was evaluated as having a high risk of bias.

#### Incomplete Outcome Data

This domain assesses whether there was any missing data and how it was handled. Fourteen studies were assessed to have a low risk of bias. Three [25,41,53] of these studies reported that no missing data emerged. One study [45] reported the usage of baseline measures in place of missing posttreatment or follow-up data. One study [46] claimed that its missing item values were replaced with the mean value of that participant’s scale scores. Another 9 studies [38-40,43,47,48,51,54,55] reported low attrition rates with balanced numbers across groups. Four studies [42,44,49,56] were assessed as high risk of bias because of high attrition rates after being randomized. Two studies [50,52] did not report any information about the attrition rate, which was assessed to be at unclear risk of bias.

#### Selective Reporting

This domain evaluates whether the study reported all the outcomes that were planned at the outset. One study [49] was assessed to be at high risk of bias, as it mentioned that nonsignificant results were not reported. A total of 19 studies [25,38-48,50-56] were assessed as having a low risk of bias.

#### Other Potential Sources of Bias

**Figure 2.**
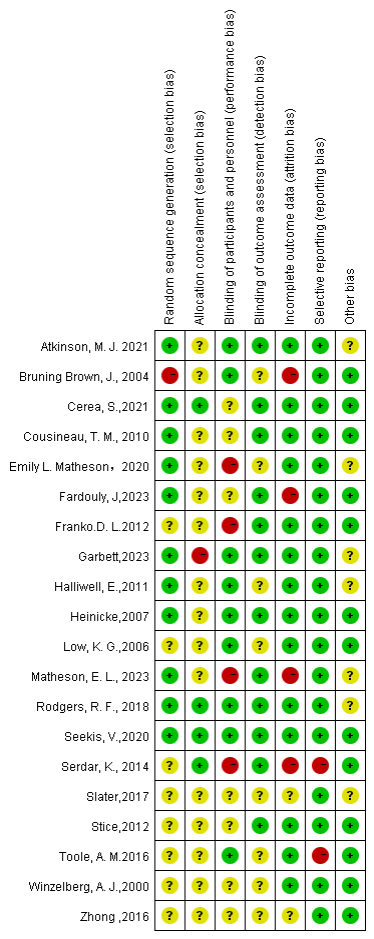
Risk of bias graph [25,38-45,47-56].

This domain assesses other potential biases not covered by the previous domains. Seven studies [25,39,40,42,52,54,55] with microinterventions lasting less than a week were assessed as unclear risk of bias. The rest appeared to be free of other biases (Figure 2) [25,38-45,47-56]. A funnel plot of SE against SMD was generated for 2 outcomes with at least 10 studies: thin-ideal internalization and body dissatisfaction. A visual inspection of the plot for the 2 funnel plots revealed the presence of publication bias (Multimedia Appendix 3).

#### Results of Syntheses

##### The Effect of Digital Interventions on Body Image Satisfaction or Dissatisfaction

**Figure 3.**
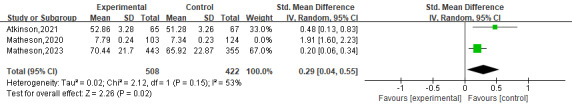
The effect of digital interventions on body satisfaction [40,42,54].

Three studies [40,42,54] reported the original data of body satisfaction among 1157 children and emerging adults by using the body satisfaction subscale of Visual Analogue Scale (VAS), and the higher scores indicated higher body satisfaction. The effect of digital interventions on body satisfaction was shown to be nonsignificant in the random-effect model (SMD=0.86, 95% CI −0.17 to 1.89) with a high level of heterogeneity across studies (*I^2^*=98%; *P*=.10). This may be due to a diverse rating system, that is, one study used the mean score for each item, while another two studies used a summed score. While the heterogeneity reduced (*I*^2^=53%) after one study [40] was excluded, and the result was a statistically significant conclusion with a small effect size (SMD=0.29, 95% CI 0.04 to 0.55; *P*=.02; Figure 3 [40,42,54]).

**Figure 4.**
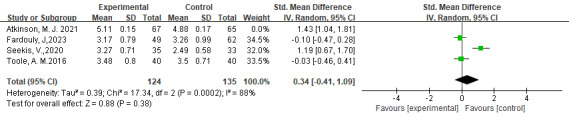
The effect of digital interventions on body appreciation [51,53,54,56].

Four studies [51,53,54,56] assessed the effect of digital interventions on body appreciation among 391 children and emerging adults. The pooled analysis showed a nonsignificant improvement in body appreciation between groups in the random-effects model with a high level of heterogeneity (SMD=0.62, 95% CI −0.19 to 1.43; *I^2^*=93%; *P*=.13). When excluding the study [54] with the outlying effect size, the overall effect did not change (SMD=0.34, 95% CI −0.41 to 1.09; *P*=.038; Figure 4 [51,53,54,56]), and the heterogeneity remained high (*I*^2^=88%).

**Figure 5.**
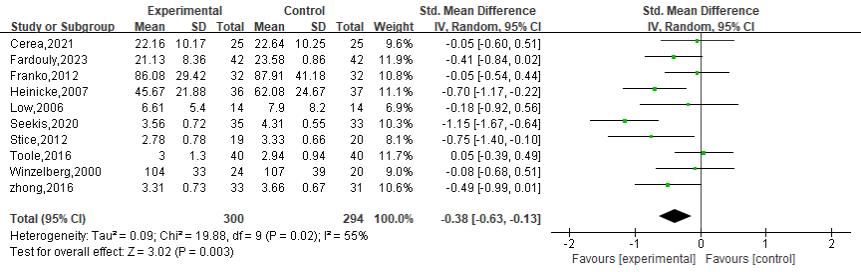
The effect of digital interventions on body dissatisfaction [41,43,45-48,50,51,53,56].

Ten RCTs [41,43,45-48,50,51,53,56] assessed the effect of digital interventions on body dissatisfaction among 594 participants, and the higher scores of the scales used in these studies indicated higher body dissatisfaction. The effect of digital interventions on body dissatisfaction was shown to be statistically significant with a small effect size in the random-effect model (SMD=−0.38, 95% CI: −0.63 to −0.13; *I*^2^=55%; *P*=.003; Figure 5 [41,43,45-48,50,51,53,56]).

**Figure 6.**
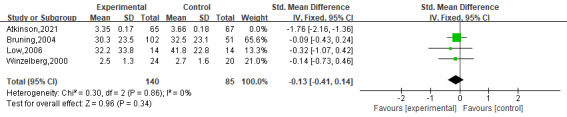
The effect of digital interventions on shape and weight concerns [43-45,54].

Four RCTs [43-45,54], including 357 participants, reported the effectiveness of digital interventions on the shape and weight concerns. Shape and weight concern is a facet of negative body image, and higher scores on the scales in the included studies reflected greater body dissatisfaction and less body image satisfaction. The pooled analysis showed non-significant improvement (SMD=−0.59, 95% CI −1.5 to 0.32) in shape and weight concern with high heterogeneity (*I*^2^=93%; *P*=.21). There was no heterogeneity (*I*^2^=0%) after one study [54] was excluded (SMD=−0.13, 95% CI −0.41 to 0.14; *P*=.34; Figure 6 [43-45,54]). However, the result was not significantly different between groups.

**Figure 7.**
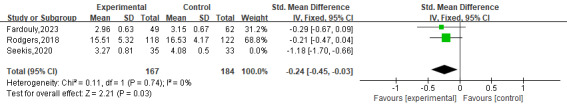
The effect of digital interventions on physical appearance comparison [39,53,56].

Three RCTs [39,53,56] measured the effects of digital interventions on physical appearance comparison among 419 participants. The higher the scores of the scales included in the studies, the more severe the physical appearance comparison among the participants. The result showed a statistically significant conclusion with high heterogeneity (SMD=−0.52, 95% CI −1.02 to −0.01; *I*^2^=82%*; P*=.004). The heterogeneity decreased to low with a small effect size (SMD=−0.24, 95% CI −0.45 to −0.03; *I*^2^=0%; *P*=.03; Figure 7 [39,53,56]) when one study [53] was excluded.

##### Effect of Digital Interventions on Internalized Outcomes

**Figure 8.**
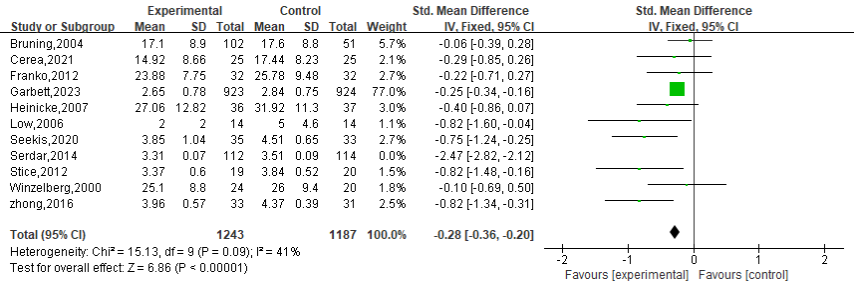
The effect of digital interventions on thin-ideal internalization [41,43-50,53,55].

Eleven RCTs [41,43-50,53,55] measured the effect of digital interventions on thin-ideal internalization among 3362 participants. The higher the scores of thin-ideal internalization, the stronger the desire to be thinner, not objectively thin. The pooled analysis showed a significant improvement in thin-ideal internalization in the random-effect model (SMD=−0.64, 95% CI −1.09 to −0.18) with high heterogeneity (*I*^2^=94; *P*<.001). The heterogeneity reduced to moderate (*I*^2^=41%) when one study [49] was excluded, while the result remained a small and significant effect size (SMD=−0.28, 95% CI −0.36 to −0.20; *P*=.001; Figure 8 [41,43-50,53,55]).

Six RCTs [25,38,39,42,49,55] measured the effectiveness of digital interventions on self-esteem among 3632 participants. Higher scores for self-esteem indicate higher body image satisfaction. The result depicted self-esteem in the experimental group was significantly better than the control group with high heterogeneity (SMD=0.28, 95% CI 0.08-0.47; *I*^2^=81%; *P*=.005). When excluding the study [49], the intervention effect on self-esteem remained significant with a small effect size (SMD=0.14, 95% CI 0.07-0.21) with low heterogeneity (*I*^2^=21%, *P*<.001; Multimedia Appendix 4).

Meta-analysis results on 3 studies [39,51,53] with 385 participants showed that self-compassion in the experimental group was significantly better than the control group with a high level of heterogeneity (SMD=0.73, 95% CI 0.25-1.2; *I*^2^=76%; *P*=.003). The heterogeneity reduced to moderate (*I*^2^=35%) after excluding the study [53], and the intervention effect on self-compassion remained significant with a medium effect size (SMD=0.55, 95% CI 0.33-0.78; *P*=.001; Multimedia Appendix 5).

Two RCTs [52,56] assessed the effect of digital interventions on self-objectification among 191 participants. Self-objectification refers to conceptualizing one’s own body as objects to be scrutinized by others, and higher levels of self-objectification indicate greater body dissatisfaction. The pooled analysis showed that self-objectification in the experimental groups was not significantly different from the control groups (SMD=−0.05, 95% CI −0.33 to 0.24; *I*^2^=0%; *P*=.75; Multimedia Appendix 6).

##### Effect of Digital Interventions on Negative Affect

Four RCTs [39,42,55,56] assessed the effect of digital interventions on negative affect among 2981 participants. The negative effects include afraid, upset, shame, hostility, and misery, and higher scores of negative affect indicate higher body dissatisfaction. The pooled analysis showed that the negative affect in the experimental groups was not superior to the control groups (SMD=−0.07, 95% CI −0.14 to 0.00; *I*^2^=0%; *P*=.05; Multimedia Appendix 7).

Four RCTs reported the effect of digital interventions on depression, anxiety, and stress among 342 participants by using the Beck Depression Inventory (BDI) [46,48], the Depression Anxiety Stress Scale-21 (DASS-21) [41], and the Social Appearance Anxiety Scale (SAAS) [53]. The higher scores in the 3 scales indicate higher depression, anxiety, and stress. Owing to the assessment of different psychological outcomes, the data could not be synthesized in the 2 studies [41,53], and there was no statistically significant improvement found in the 2 studies, separately. However, the pooled analysis of the BDI group showed a significant improvement in depression in the fixed-effects model with a medium effect size (SMD=−0.59, 95% CI −0.97 to −0.21; *I*^2^=0%; *P*=.002; Multimedia Appendix 8).

##### Effect of Digital Interventions on Eating Behaviors

Four RCTs [41,44,45,49] assessed the effect of digital interventions on eating disorders among 457 participants. The pooled analysis of eating disorders showed there was no statistically significant difference between groups (SMD=−0.37, 95% CI −1.36 to 0.6) with high heterogeneity (*I*^2^=95%; *P*=.45). The heterogeneity decreased to low (*I*^2^=0%) after one study [49] was excluded, and the result remained no significant difference between groups (SMD=−0.04, 95% CI −0.31 to 0.22; *P*=.75; Multimedia Appendix 9).

Two RCTs [46,48] assessed the effect of digital interventions on eating restraint among 203 participants. Higher scores of eating restraint are indicative of greater use of extreme weight loss behaviors. The pooled analysis of eating restraint showed that there was no statistically significant difference between groups (SMD=−0.36, 95% CI −0.71 to 0.00; *I*^2^=31%; *P*=.05; Multimedia Appendix 10).

## Discussion

### Principal Findings

This systematic review and meta-analysis synthesized evidence from 20 RCTs involving 5251 participants to evaluate the effectiveness of digital interventions (eg, web pages, mobile apps, and social media) on body dissatisfaction and related outcomes. Key findings indicated that digital interventions significantly improved body dissatisfaction, physical appearance comparison, thin-ideal internalization, self-esteem, self-compassion, and depression, with small to medium effect sizes. However, there was substantial heterogeneity across studies, and potential publication bias was detected. Digital interventions did not significantly improve negative affect (including depression and anxiety) and eating behaviors.

### Study Characteristics

All the included studies have been conducted in the last 2 decades, but only 2 studies have been done in developing countries. This indicates that high-quality RCTs need to be conducted further in this area, especially to explore their effectiveness on body dissatisfaction in different ethnic and cultural backgrounds in developing countries [57]. Ten out of twenty included studies selected different theories or models to motivate participants to reduce the pursuit of thin-ideal internalization, to improve emotional regulation, such as self-compassion, and to develop coping strategies with the involvement of parents, peers, and the media. The cognitive dissonance theory, cognitive behavioral theory, and sociocultural theory were recognized as the most widely mentioned theories to reduce body dissatisfaction in the studies. The average sample size was high 200s, but most studies included participants of less than 100. This would result from the narrow age range and the diagnosis of anorexia nervosa and other eating or psychiatric disorders among children and emerging adults in the inclusion criteria. The participants’ ages ranged from 7 to 25 years old, and 6 studies included those younger than 14 years old in private or public schools as participants. Nearly 80% of participants were females in this review, and 9 studies only included female college students or undergraduates as participants, which accounted for 16.7% of the total participants. This can indicate that females would be more likely to experience body dissatisfaction, like other studies [58].

### Comparison to Previous Work

The meta-analysis revealed that digital interventions showed a small to medium effect across most outcomes in body satisfaction and internalized outcomes, and the result was similar to another systematic review [24]. In the systematic review, although there was no quantitative synthesis, body dissatisfaction, body esteem, body appreciation, and other body image outcomes were also included in the review to explore the effectiveness of digital interventions in emerging adults and emerging adults. The results showed 8 out of 15 studies reported digital interventions were effective in improving at least one body image outcome from pre-post interventions with mostly small to medium effect sizes. This means digital interventions are superior to some traditional interventions, such as brochures, documentaries, newspapers, or magazines. It may result from the advantages of digital interventions, such as real-time feedback and motivation enhancement [59], and anonymity of platforms [60], which could reduce the shame and social anxiety emerging adults face when dealing with body image-related issues. However, given that effect sizes were small to medium, it would be caused by the following reasons. First, the intervention duration of this meta-analysis was 6 weeks on average. Second, digital intervention had its limitations, such as being incapable of genuinely caring about one’s feelings during the intervention process. This indicated that digital interventions with an in-person element would be associated with greater effectiveness in body image [61].

Digital interventions did not significantly reduce the negative affect (including depression and anxiety) and eating behaviors. However, another 2 systematic reviews [61,62] found a significant effect of digital interventions on depression and anxiety among emerging adults and emerging adults. This would result from the contents of the digital interventions. The digital interventions of the 2 systematic reviews specifically targeted mental health disorders, including depression and anxiety. However, this meta-analysis mainly targeted improving body dissatisfaction with depression and anxiety as secondary outcomes in some studies included. Furthermore, it indicated that digital psychotherapy did not significantly reduce both eating disorders and restrained eating in this meta-analysis. This would be caused by the following reasons. First, no more than 4 RCTs were included in this meta-analysis to explore the effects of digital interventions on eating disorders or restraint eating. Second, the age of participants in this meta-analysis ranged from 7 to 25 years. Especially for the emerging adults who were predominant participants, who have already established eating behavior habits, which would be so ingrained and resistant to change through short-term digital psychological therapy. According to the Transtheoretical Model [63], the process of behavior change is complicated with 6 different stages, and it would not be easy to change eating behaviors. In addition, the digital interventions in the 20 included RCTs were aimed at addressing body dissatisfaction rather than specific eating disorder symptoms, which might account for the insignificant outcome in changing eating behaviors.

### Implications for Clinical Practice

The findings suggest that digital interventions can serve as scalable and cost-effective supplementary tools to improve body dissatisfaction in children and emerging adults. Clinicians may recommend digital interventions as adjuncts to traditional face-to-face therapies, particularly for individuals with limited access to in-person mental health services due to cost, stigma, or geographic barriers. Body dissatisfaction, thin-ideal internalization, eating disorders, and negative affect should be addressed simultaneously in a single intervention to verify the effectiveness of digital interventions in the future. Future studies should be conducted in Asia and some other low-income countries, as this review found that studies in these regions are lacking. Furthermore, the quality of included studies held room for improvement.

### Limitations

This systematic review has several limitations. First, the high heterogeneity in meta-analysis may result from the use of different scales to assess the same outcomes, even using different versions or varying score systems (eg, summed versus mean scores) of the same scales. However, the SMD was selected to express the size of the intervention effect, instead of the mean difference. Second, confidence in the meta-analysis was limited because waitlist groups were set in 7 studies, and assessment-only control (receive no intervention) groups were set in another 4 studies, rather than active control groups. Third, there was insufficient data on long‐term follow‐up and a potential gap that future RCTs can look to fill. Specific tactics could be analyzed to maintain engagement in body image interventions for children and emerging adults, such as integrating digital interventions into existing curricula. Fourth, the quality of included studies held room for improvement, with most of the RCTs included being rated as “unclear risk” for bias. Fifth, publication bias may have influenced the results, particularly for outcomes with small sample sizes, highlighting the need for larger, registered RCTs.

### Conclusion

Digital interventions could help children and emerging adults improve body satisfaction or dissatisfaction, physical appearance comparison, thin-ideal internalization, self-esteem, self-compassion, and depression. Because of the limitations, the results should be generalized with caution. In the future, high-quality RCTs with longer intervention duration and long-term follow-up should be conducted, especially in different races and cultures in transitional countries.

## Supplementary material

10.2196/72231 Multimedia Appendix 1 Search strategy.

10.2196/72231 Multimedia Appendix 2 Study quality assessment.

10.2196/72231 Multimedia Appendix 3 Funnel plot.

10.2196/72231 Multimedia Appendix 4 The effect of digital interventions on self-esteem [25,38,39,42,49,55].

10.2196/72231 Multimedia Appendix 5 The effect of digital interventions on self-compassion [39,51,53].

10.2196/72231 Multimedia Appendix 6 The effect of digital interventions on self-objectification [52,56].

10.2196/72231 Multimedia Appendix 7 The effect of digital interventions on negative affect [39,42,55,56].

10.2196/72231 Multimedia Appendix 8 The effect of digital interventions on depression [41,53].

10.2196/72231 Multimedia Appendix 9 The effect of digital interventions on eating disorders [41,44,45,49].

10.2196/72231 Multimedia Appendix 10 The effect of digital interventions on eating restraint [46,48].

10.2196/72231 Checklist 1 PRISMA 2020 checklist.

## Abbreviations

BDI: Beck Depression Inventory
BIS: behavior interventions and supports
CBT: cognitive-behavioral therapy
CNKI: China National Knowledge Infrastructure
DASS-21: Depression Anxiety Stress Scale-21
EBSCO: Elton B. Stephens Company
PICOS: population, intervention, comparison, outcome, and study design
PRISMA: Preferred Reporting Items for Systematic Reviews and Meta-Analyses
RCT: randomized controlled trial
SAAS: Social Appearance Anxiety Scale
SMD: standardized mean difference
VAS: Visual Analogue Scale
