# Supplementary material for: Digital Interventions for Improving Body Dissatisfaction in Children and Emerging Adults: Systematic Review and Meta-Analysis
Source: Interact J Med Res. 2025 Aug 13;14:e72231. doi: 10.2196/72231 (PMC12345061; doi:10.2196/72231)
Supplement: Multimedia Appendix 1 [file ijmr-v14-e72231-s001.doc]

**Appendix 1:** Search strategy

**PubMed, Web of Science, MEDLINE, EBSCO, and Cochrane Library**

# 1: youth OR young* OR child* OR adolescent OR teen* OR juvenile OR junior OR girl OR boy OR adult or students

# 2: remote OR website OR digital OR online OR network OR phone OR internet OR eHealth OR mHealth OR application OR multimedia OR social media OR zoom OR Facebook OR Instagram OR telephone OR virtual reality

# 3: (body image) OR (body shape) OR (body dissatisfaction) OR (body weight) OR (physical appearance)

#4 (Type of Study): randomized controlled trials.

The final search strategy is #1 AND #2 AND #3, with #4 applied as a filter.

CNKI, WANFANG

# 1: 儿童 OR青少年 OR 年轻人 OR 学生 OR 未成年 OR青年

# 2: 数字化 OR数智化 OR远程 OR手机 OR视频 OR社交媒体OR 电话OR 线上

# 3: 身体意象 OR身体形象 OR体象 OR身体自我
