# Supplementary material for: Digital Interventions for Improving Body Dissatisfaction in Children and Emerging Adults: Systematic Review and Meta-Analysis
Source: Interact J Med Res. 2025 Aug 13;14:e72231. doi: 10.2196/72231 (PMC12345061; doi:10.2196/72231)
Supplement: Multimedia Appendix 2 [file ijmr-v14-e72231-s002.doc]

| **Study** | **Random sequence generation (Selection bias)** | **Allocation concealment (Selection bias)** | **Blinding of participants and personnel (Performance bias)** | **Blinding of outcome assessment (Detection bias)** | **Incomplete outcome data (Attrition bias)** | **selective reporting (Reporting bias)** | **Other potential sources of bias** | **Overall**  **Risk of bias** |
| --- | --- | --- | --- | --- | --- | --- | --- | --- |
| Winzelberg et al [43] | Unclear risk: only mentioned "participants were randomly assigned to Student Bodies or a delayed-intervention control group", no specific randomized allocation plan was mentioned. | *Unclear risk: Allocation concealment was not mentioned in the text.* | Unclear risk: No blinding procedures described | Unclear risk: No description of how information was concealed from researchers | Low risk: baseline n=60, 8 dropped out during the pre-post intervention period. An additional 8 participants (3 intervention, 5 control) did not complete the 3-month follow-up. | Low risk: All outcomes appear to be reported on | Low risk | High risk |
| Bruning Brown et al [44] | High risk: Students were assigned to groups based on class schedule rather than randomization. | *Unclear risk: Allocation concealment was not mentioned in the text.* | Low risk: To reduce potential cross-contamination, students in the intervention were instructed not to discuss it with other students | Unclear risk: No description of how information was concealed from researchers | High risk:50% attrition in the intervention group parents | Low risk: All outcomes appear to be reported on | Low risk | High risk |
| Low et al [45] | Unclear risk: only mentioned "after baseline assessment, women were randomized into four groups", specific allocation not mentioned. | *Unclear risk: Allocation concealment was not mentioned in the text.* | Low risk: participants using a pseudonym, online-delivered, can be seen as blinding of personnel. | Unclear risk: No description of how information was concealed from researchers | Low risk: 72 original participants, 7 non-compliers (9.7%), 4 participants could not be located at long-term follow-up, resulting in 6% attrition rate. Using baseline measures in place of missing post-treatment or follow-up data. | Low risk: All outcomes appear to be reported on | Low risk | High risk |
| Heinicke et al [46] | Low risk: Girls were randomly assigned by a computer-generated randomization plan. | *Unclear risk: Allocation concealment was not mentioned in the text.* | Low risk: "Therapist did not see the participant face-to-face", can be viewed as blinding to researchers. | Low risk: online assessment, questionnaires were completed online by participants. | Low risk: Little missing data on returned questionnaires (less than 1%), and appeared to be random. Missing item values were replaced with the mean value of that participant’s scale score. | Low risk: All outcomes appear to be reported on. | Low risk | Moderate risk |
| Cousineau (2010) [38] | Low risk: Block randomization, "students were randomized to groups by gender." | *Unclear risk: Allocation concealment was not mentioned in the text.* | Unclear risk: No blinding procedures described | Low risk: assessment online. | Low risk: low attrition rate. Complete data were available for 178 out of 190 enrolled participants (94%). | Low risk: All outcomes appear to be reported on | Low risk | Moderate risk |
| Halliwell (2011) [25] | Low risk: Randomization table," Randomly allocated through a computer-generated randomization table (www.randomization.com)" | *Unclear risk: Allocation concealment was not mentioned in the text.* | Low risk: Parents were asked to refrain from discussing the details of the study with their daughter until it had been completed, girls were asked not to talk to each other during the first part. | Unclear risk: No description of how information was concealed from researchers | Low risk: There does not appear to have any drop out- at least these are not reported on. | Low risk: All outcomes appear to be reported on | Unclear risk: A short intervention period (<1 week) | High risk |
| Franko et al [47] | Unclear risk: Only mentioned " Participants were randomly assigned to either the intervention or control condition", the specific allocation plan was not mentioned | *Unclear risk: Allocation concealment was not mentioned in the text.* | High risk: “it is not likely that the control websites were interpreted as treatment, because they focused on science”，can be seen as not blinded to the participants. | Low risk: sent questionnaire packet by mail. questionnaires were completed by mail. | Low risk: 20% attrition rates, (80%) completed all three assessments. | Low risk: All outcomes appear to be reported on. | Low risk | High risk |
| Stice et al [48] | Unclear risk: Only mentioned "participants were randomly assigned ", the specific allocation plan was not mentioned | *Unclear risk: Allocation concealment was not mentioned in the text.* | Unclear risk: No blinding procedures described | Low risk: Female assessors were blinded to the participants' condition. | Low risk: Low attrition rates: at baseline n=107, post-test n=105, follow-up n=105. | Low risk: All outcomes appear to be reported on. | Low risk | High risk |
| Serdar et al [49] | Unclear risk: only mentioned "participants were randomized and notified via email of their inclusion status and group assignment. "A specific allocation plan was not mentioned. | *Low risk: participants were notified via email* | High risk: Participants were not blinded, "notified via email of their inclusion status and group assignment." | Low risk: Group members emailed de-identified homework to the research coordinator. | High risk: High attrition rates (nearly 40%), pre-test n=333, post-test n=199 | High risk: Nonsignificant results are not reported here. | Low risk | High risk |
| Zhong et al [50] | **Unclear risk**: No mention of randomized allocation | **Unclear risk**: Allocation concealment was not mentioned in the text. | **Unclear risk**: No blinding procedures described | **Unclear risk**: No description of how information was concealed from researchers | **Unclear risk**: No information provided on dropouts or attrition. | **Low risk**: All outcomes appear to be reported on. | **Low risk** | **High risk** |
| Toole et al [51] | Unclear risk: only mentioned "participants were randomly assigned to either the intervention or waitlist control group", no specific allocation plan was mentioned. | Unclear risk: Allocation concealment was not mentioned in the text. | Low risk: Participants completed the self-report measures on a computer. | Unclear risk: No description of how information was concealed from researchers | Low risk: 4.6% Attrition rate | Low risk: All outcomes appear to be reported on | Low risk | High risk |
| Slater et al [52] | Unclear risk: Only mentioned "participants were randomly assigned ", the specific allocation plan was not mentioned | Unclear risk: Allocation concealment was not mentioned in the text. | Unclear risk: No blinding procedures described | Unclear risk: No description of how information was concealed from researchers | Unclear risk: No information provided on dropouts or attrition. | Low risk: All outcomes appear to be reported on. | Unclear risk: A short intervention period (<1 week) | High risk |
| Rodgers et al [39] | Low risk: Randomization schedule, "randomly allocated to the control or experimental group by using a randomization schedule." | Low risk: via email. | Low risk: blinded to participants (The assessments were housed on a survey software, separate from the app, and the procedure was identical for participants in both the intervention and the control group) | Low risk: assessment online. | Low risk: Low attrition rates, baseline n = 274, n = 251 (92%) completed the assessments at Time 2, and n = 237 (86%) at Time 3. | Low risk: All outcomes appear to be reported on | Unclear risk: A short intervention period (<1 week) | Low risk |
| Matheson et al [40] | Low risk: Qualtrics, "Children were randomly assigned to one of three viewing conditions using the randomization by minimization function in Qualtrics." | Unclear risk: Allocation concealment was not mentioned in the text. | High risk: This was a school study; it is unlikely that students were blinded to other conditions. | Unclear risk: No description of how information was concealed from researchers | Low risk: Attrition was minimal (8/1329 =.006%), but unclear if this was for both genders and age groups | Low risk: All outcomes appear to be reported on | Unclear risk: A short intervention period (<1 week) | High risk |
| Seekis et al [53] | Low risk: cluster randomization," participants were assigned to either the intervention group or waitlist control group via cluster randomization “ | Low risk: participants were invited, by email, to join a closed Facebook group by the facilitator. | Low risk: Participants assigned to the waitlist control group were informed that the study was about the importance of understanding the type of strategies used by women with body concerns. | Low risk: online assessment questionnaires were completed by participants online. | Low risk: There was no missing data | Low risk: All outcomes appear to be reported on | Low risk | Low risk |
| Atkinson et al [54] | Low risk: quasi-random allocation, "Whole timeslots were randomly allocated to a condition in an open computer laboratory, representing quasi-random allocation" | Unclear risk: Allocation concealment was not mentioned in the text. | Low risk: All assessments were self-reported anonymously via computer. | Low risk: All assessments were self-reported anonymously via computer. | Low risk: Low attrition rates, at baseline n=202, post-test = 192, follow-up = 190. | Low risk: All outcomes appear to be reported on | Unclear risk: A short intervention period (<1 week) | Moderate risk |
| Cerea et al [41] | Low risk: Block randomization, participants were randomized into two groups | Low risk: contacted by e-mail | Unclear risk: No blinding procedures described | Low risk: online questionnaires, all the assessments were done online. | Low risk: no missing data emerged. | Low risk: All outcomes appear to be reported on | Low risk | Low risk |
| Garbett et al [55] | Low risk: Block randomization, using an automated web-based randomizer, was performed with a 1:1 allocation in blocks of 4, 6, and 8. | High risk: Participants and researchers were not concealed from the randomized arm. | Low risk: "All communication between the research agency and the participants took place on the web via WhatsApp." can be seen as blinding of participants and personnel. | Low risk: Data analysts will be blinded to the condition throughout | Low risk: Low attrition rate, at baseline n=1855, post-test n=1765, follow-up n=1789. | Low risk: All outcomes appear to be reported on. | Unclear risk: A short intervention period (<1 week) | Moderate risk |
| Matheson et al [42] | **Low risk**: computer software," randomization scheme was generated by a research agency using a validated computer software." | **Unclear risk**: Allocation concealment was not mentioned in the text. | **High risk**: Blinding of the participants was not possible. | **Low risk**: Data analysts will be blinded to condition (dummy coded). | **High risk**: Attrition was high (531/858, 61.9%) | **Low risk**: All outcomes appear to be reported on | **Unclear risk:** A short intervention period (<1 week) | **High risk** |
| Fardouly et al [56] | **Low risk**: Qualtrics," participants were randomly assigned via a secure survey platform (i.e., Qualtrics)" | **Unclear risk**: Allocation concealment was not mentioned in the text. | **Unclear risk**: No blinding procedures described | **Low risk**: participants were sent the pre-post survey via email. | **High risk**: High attrition rates, baseline n=221, post-test n=159, follow-up n=120 | **Low risk**: All outcomes appear to be reported on | **Low risk** | **High risk** |

43. Winzelberg AJ, Eppstein D, Eldredge KL, Wilfley D, Dasmahapatra R, Dev P, Taylor CB: **Effectiveness of an Internet-based program for reducing risk factors for eating disorders.** *Journal of consulting and clinical psychology* 2000, **68:**346-350.

44. Bruning Brown J, Winzelberg AJ, Abascal LB, Taylor CB: **An evaluation of an Internet-delivered eating disorder prevention program for adolescents and their parents.** *J Adolesc Health* 2004, **35:**290-296.

45. Low KG, Charanasomboon S, Lesser J, Reinhalter K, Martin R, Jones H, Winzelberg A, Abascal L, Taylor CB: **Effectiveness of a computer-based interactive eating disorders prevention program at long-term follow-up.** *Eat Disord* 2006, **14:**17-30.

46. Heinicke BE, Paxton SJ, McLean SA, Wertheim EH: **Internet-delivered targeted group intervention for body dissatisfaction and disordered eating in adolescent girls: a randomized controlled trial.** *J Abnorm Child Psychol* 2007, **35:**379-391.

38. Cousineau TM, Franko DL, Trant M, Rancourt D, Ainscough J, Chaudhuri A, Brevard J: **Teaching adolescents about changing bodies: Randomized controlled trial of an Internet puberty education and body dissatisfaction prevention program.** *Body image* 2010, **7:**296-300.

25. Halliwell E, Easun A, Harcourt D: **Body dissatisfaction: can a short media literacy message reduce negative media exposure effects amongst adolescent girls?** *Br J Health Psychol* 2011, **16:**396-403.

47. Franko DL, Jenkins A, Rodgers RF: **Toward Reducing Risk for Eating Disorders and Obesity in Latina College Women.** *Journal of Counseling & Development* 2012, **90:**298-307.

48. Stice E, Rohde P, Durant S, Shaw H: **A preliminary trial of a prototype Internet dissonance-based eating disorder prevention program for young women with body image concerns.** *J Consult Clin Psychol* 2012, **80:**907-916.

49. Serdar K, Kelly NR, Palmberg AA, Lydecker JA, Thornton L, Tully CE, Mazzeo SE: **Comparing online and face-to-face dissonance-based eating disorder prevention.** *Eat Disord* 2014, **22:**244-260.

50. Yunyu Z: **Towards Evaluating an Internet-Based Dissonance Intervention to Ｒeduce Body Dissatisfaction Among Young Chinese Women: Some Preliminary Findings.** *Community Psychology Research* 2016, 2:150-167.

51. Toole AM, Craighead LW: **Brief self-compassion meditation training for body image distress in young adult women.** *Body Image* 2016, **19:**104-112.

52. Slater A, Halliwell E, Jarman H, Gaskin E: **More than Just Child's Play?: An Experimental Investigation of the Impact of an Appearance-Focused Internet Game on Body Image and Career Aspirations of Young Girls.** *J Youth Adolesc* 2017, **46:**2047-2059.

39. Rodgers RF, Donovan E, Cousineau T, Yates K, McGowan K, Cook E, Lowy AS, Franko DL: **BodiMojo: Efficacy of a Mobile-Based Intervention in Improving Body Image and Self-Compassion among Adolescents.** *J Youth Adolesc* 2018, **47:**1363-1372.

40. Matheson EL, Lewis-Smith H, Diedrichs PC: **The effectiveness of brief animated films as a scalable micro-intervention to improve children's body image: A randomised controlled trial.** *Body Image* 2020, **35:**142-153.

53. Seekis V, Bradley GL, Duffy AL: **Does a Facebook-enhanced Mindful Self-Compassion intervention improve body image? An evaluation study.** *Body Image* 2020, **34:**259-269.

54. Atkinson MJ, Diedrichs PC: **Examining the efficacy of video-based microinterventions for improving risk and protective factors for disordered eating among young adult women.** *Int J Eat Disord* 2021, **54:**708-720.

41. Cerea S, Ghisi M, Bottesi G, Manoli T, Carraro E, Doron G: **Cognitive Behavioral Training Using a Mobile Application Reduces Body Image-Related Symptoms in High-Risk Female University Students: A Randomized Controlled Study.** *Behav Ther* 2021, **52:**170-182.

55. Garbett KM, Haywood S, Craddock N, Gentili C, Nasution K, Saraswati LA, Medise BE, White P, Diedrichs PC, Williamson H: **Evaluating the Efficacy of a Social Media-Based Intervention (Warna-Warni Waktu) to Improve Body Image Among Young Indonesian Women: Parallel Randomized Controlled Trial.** *J Med Internet Res* 2023, **25:**e42499.

42. Matheson EL, Smith HG, Amaral ACS, Meireles JFF, Almeida MC, Linardon J, Fuller-Tyszkiewicz M, Diedrichs PC: **Using Chatbot Technology to Improve Brazilian Adolescents' Body Image and Mental Health at Scale: Randomized Controlled Trial.** *JMIR Mhealth Uhealth* 2023, **11:**e39934.

56. Fardouly J, Slater A, Parnell J, Diedrichs PC: **Can following body positive or appearance neutral Facebook pages improve young women's body image and mood? Testing novel social media micro-interventions.** *Body Image* 2023, **44:**136-147.
