# Supplementary figures and images for: Digital Interventions for Improving Body Dissatisfaction in Children and Emerging Adults: Systematic Review and Meta-Analysis
Source: Interact J Med Res. 2025 Aug 13;14:e72231. doi: 10.2196/72231 (PMC12345061; doi:10.2196/72231)

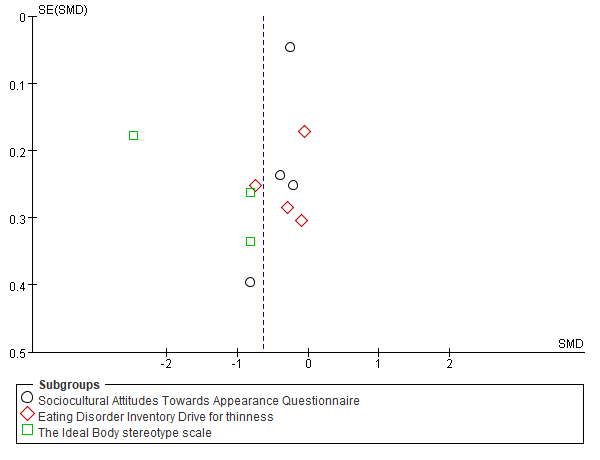


Funnel plot: thin-ideal internalization


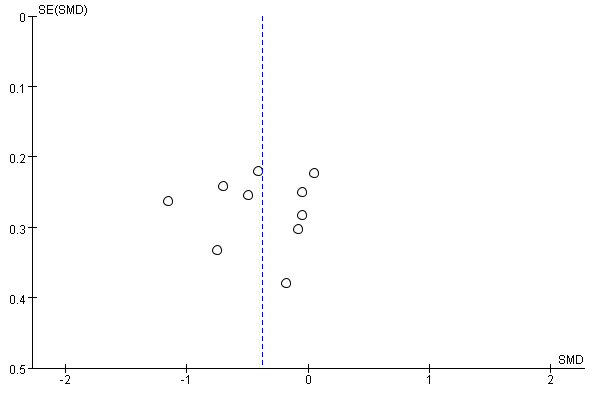


Funnel plot: Body dissatisfaction

Supplement: Multimedia Appendix 3 [file ijmr-v14-e72231-s003.doc]

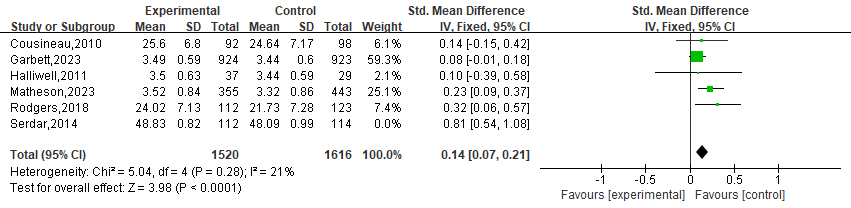

Supplement: Multimedia Appendix 4 [file ijmr-v14-e72231-s004.doc]

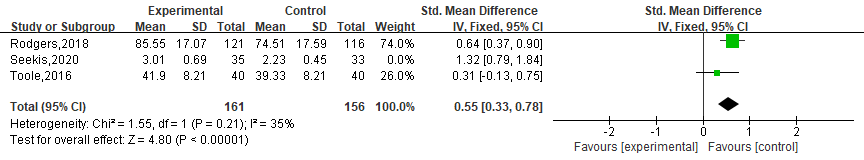

Supplement: Multimedia Appendix 5 [file ijmr-v14-e72231-s005.doc]

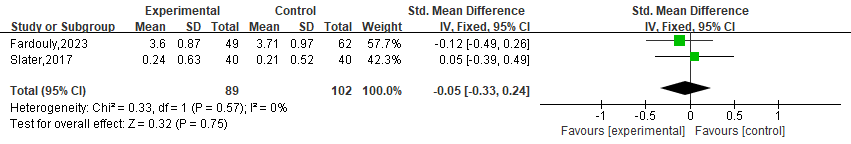

Supplement: Multimedia Appendix 6 [file ijmr-v14-e72231-s006.doc]

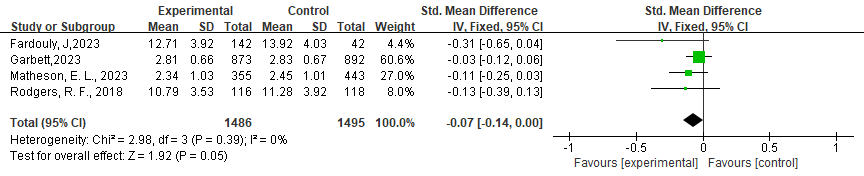

Supplement: Multimedia Appendix 7 [file ijmr-v14-e72231-s007.doc]

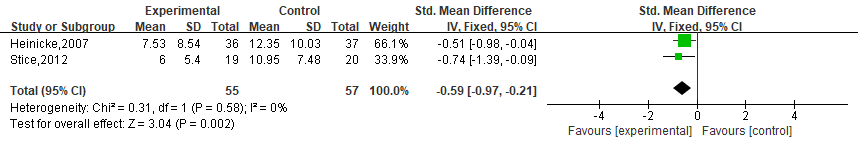

Supplement: Multimedia Appendix 8 [file ijmr-v14-e72231-s008.doc]

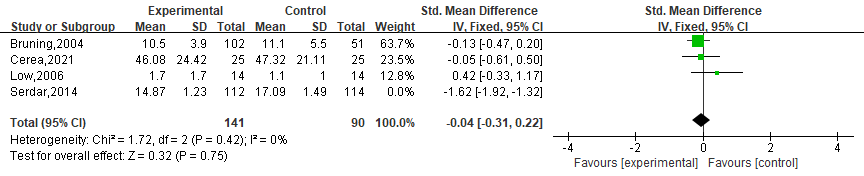

Supplement: Multimedia Appendix 9 [file ijmr-v14-e72231-s009.doc]

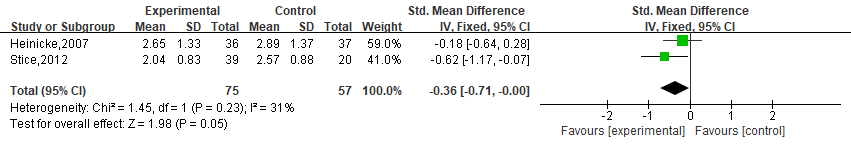

Supplement: Multimedia Appendix 10 [file ijmr-v14-e72231-s010.doc]
